# Herpesviruses reactivation following COVID-19 vaccination: a systematic review and meta-analysis

**DOI:** 10.1186/s40001-023-01238-9

**Published:** 2023-08-10

**Authors:** Arman Shafiee, Mohammad Javad Amini, Razman Arabzadeh Bahri, Kyana Jafarabady, Seyyed Amirhossein Salehi, Hamed Hajishah, Sayed-Hamidreza Mozhgani

**Affiliations:** 1 Clinical Research Development Unit, Alborz University of Medical Sciences, Karaj, Iran; 2 Student Research Committee, School of Medicine, Alborz University of Medical Sciences, Karaj, Iran; 3 Tehran University of Medical Sciences, Tehran, Iran; 4 Student Research Committee, School of Medicine, Shahid Beheshti University of Medical Sciences, Tehran, Iran; 5 Student Research Committee, Tehran Medical Sciences Branch, Islamic Azad University, Tehran, Iran; 6 Department of Microbiology, School of Medicine, Alborz University of Medical Sciences, Karaj, Iran; 7 Non-Communicable Diseases Research Center, Alborz University of Medical, Sciences, Karaj, Iran

**Keywords:** SARS-CoV-2, COVID-19, Vaccination, Herpesvirus, HHV

## Abstract

**Background:**

The reactivation of herpesviruses (HHV) in COVID-19 patients is evident in the literature. Several reports have been published regarding the reactivation of these viruses (HSV, VZV, EBV, and CMV) among those who got COVID-19 vaccines. In this study, we aimed to review the current evidence to assess whether HHVs reactivation has any association with the prior administration of COVID-19 vaccines.

**Methods:**

A systematic search was conducted on 25 September 2022 in PubMed/MEDLINE, Web of Science, and EMBASE. We included all observational studies, case reports, and case series which reported the reactivation of human herpesviruses following administration of COVID-19 vaccines.

**Results:**

Our systematic search showed 80 articles that meet the eligibility criteria. Among the evaluated COVID-19 vaccines, most of the vaccines were mRNA based. Evidence from observational studies showed the possible relation between COVID-19 vaccine administration and VZV and HSV reactivation. The results of our proportion meta-analysis showed that the rate of VZV reactivation among those who received the COVID-19 vaccine was 14 persons per 1000 vaccinations (95% CI 2.97–32.80). Moreover, our meta-analysis for HSV reactivation showed the rate of 16 persons per 1000 vaccinations (95% CI 1.06–46.4). Furthermore, the evidence from case reports/series showed 149 cases of HHV reactivation. There were several vaccines that caused reactivation including BNT162b2 mRNA or Pfizer–BioNTech (*n* = 76), Oxford-AstraZeneca (*n* = 22), mRNA-1273 or Moderna (*n* = 17), Sinovac (*n* = 4), BBIBP-CorV or Sinopharm (*n* = 3), Covaxin (*n* = 3), Covishield (*n* = 3), and Johnson and Johnson (*n* = 1). Reactivated HHVs included varicella-zoster virus (VZV) (*n* = 114), cytomegalovirus (CMV) (*n* = 15), herpes simplex virus (HSV) (*n* = 14), Epstein-Barr virus (EBV) (*n* = 6), and HHV-6 (*n* = 2). Most cases reported their disease after the first dose of the vaccine. Many patients reported having comorbidities, of which hypertension, diabetes mellitus, dyslipidemia, chicken pox, and atrial fibrillation were common.

**Conclusion:**

In conclusion, our study showed the possible association between COVID-19 vaccination and herpesvirus reactivation. The evidence for VZV and HSV was supported by observational studies. However, regarding other herpesviruses (EBV and CMV), further research especially from observational studies and clinical trials is required to elucidate the interaction between COVID-19 vaccination and their reactivation.

**Supplementary Information:**

The online version contains supplementary material available at 10.1186/s40001-023-01238-9.

## Introduction

Since late 2019, the novel severe acute respiratory syndrome coronavirus 2 (SARS-CoV-2), known as coronavirus disease 2019 (COVID-19), has brought up many concerns due to its widespread, which has led to a considerable number of studies evaluating a variety of therapeutic approaches for COVID-19, including chloroquine, ivermectin, remdesivir, nucleoside analogs, hydroxychloroquine, monoclonal antibodies, famotidine, convalescent plasma, herbal medicine, and natural compounds [1–3]. To date, utilizing vaccines is one of the most effective ways to control the pandemic. COMIRNATY (the COVID-19 mRNA vaccine BNT162b2 by BioNTech– Pfizer); COVID-19 Vaccine Moderna (mRNA-1273 by Moderna); VAXZEVRIA (ChAdOx1- nCoV19 by AstraZeneca-Oxford University); and COVID-19 Vaccine Janssen (Ad26.COV2.S by Janssen) are among the most popular vaccines used against the COVID-19 [4]. Notwithstanding the different mechanisms of action, all these vaccines that have been administered have some local and systemic side effects after the injection, such as site pain and swallowing, fever, arthralgia, headache, and vomiting [5, 6].

Herpesviridae consists of a DNA virus that falls into a varicella-zoster virus (VZV), cytomegalovirus (CMV), Epstein-Barr virus (EBV), and herpes simplex virus (HSV). Herpesviruses (HHV) are mostly known for their ability to cause latent infection, which can become reactivated by triggers such as stress, lack of sleep, physical fatigue, exposure to sunlight, fever, menstruation, and surgical resection [7]. HHVs are capable of remaining in different types of body cells after the first infection and become reactivated when the host is experiencing an immunocompromised state critically ill patients, sepsis shock, intensive care unit (ICU) administration, usage of anti-inflammatory drugs, and prolonged ventilation are risk factors for the immunocompromised state which can lead to reactivation of these viruses [8–11]. All these conditions can happen during severe and critical COVID-19. Preliminary work on the incidence of herpesvirus reactivation in COVID-19 patients was undertaken by Simmonet et al., which shows that 85% of critically ill patients with COVID-19 in ICU have developed EBV, CMV, and HHV-6 viremia [9]. It may reasonably be doubted whether the vaccination for COVID-19 can be a reason for the herpes virus’s virus family's reactivation. In this connection, VZV reactivation after vaccine administration was reported in 91 patients who were mostly represented by mild to moderate cutaneous lesions [12].

Reactivation of other HHVs (EBV, CMV, and HSV) following COVID-19 vaccination have been reported in several case reports. Taken together all these reported cases suggest that although vaccines administration rarely results in severe side effect, early diagnosis and prophylaxis would be essential for decreasing the morbidity and side effects. Therefore, the present study was designed to determine the correlation between the COVID-19 vaccine administration and reactivation of herpes virus and review the cases who have experienced this condition, to increase awareness about the clinical manifestation of herpes reactivation following COVID-19 vaccination.

## Methods

We conducted a systematic review following the Preferred Reporting Items for Systematic Reviews and Meta-Analyses (PRISMA) guidelines and guideline provided by the Cochrane Handbook for Systematic Reviews of Interventions [13, 14]. The protocol of this study was registered with the following number: IR.ABZUMS.REC.1402.116.

### Search strategy

A systematic search was conducted in several international databases including Medline (via PubMed), Embase, and Web of science up to 25 September 2022. No restrictions were applied to the search results we retrieved. Furthermore, studies that were eligible were found by evaluating the references of the papers that might be included. The Boolean operators and the following keywords were combined together to create the right approach for our comprehensive search: COVID-19, SARS-CoV-2, coronavirus, Herpesviridae, HSV, herpes simplex virus, varicella-zoster virus, VZV, Epstein-Barr virus, EBV, cytomegalovirus, CMV. Additional file 1: Table S2 provides a thorough description of the search process for each database, along with exact results and performance times.

### Eligibility criteria

Using the PICOT specification, the inclusion criteria were as follows: 1) Population: adults receiving COVID-19 vaccine either first or second dose; 2) Intervention: COVID-19 vaccines; 3) Comparison: If applicable (since most studies did not evaluate a control group), those who were not vaccinated against COVID-19; 4) Outcome: reactivation of Herpesviridae; and 5) Type of Study: Observational studies, case reports, and case series. Conference abstracts were also included. The exclusion criteria included review studies, opinion studies, and letters to the editor devoid of any relevant info.

### Screening and data extraction

The papers were initially screened by title and abstract, and then the full texts were screened. Discussions were used to settle disagreements. A spreadsheet in Excel was used to extract the data. The extracted for observational studies were Author, Year, Country, Type of study (Registry/ Duration), Population, Total patients, Vaccine, Reactivated virus, and Main Findings of each cohort. For case reports/series we extracted Author, Year, Country, Total Patients, Age, Vaccine, Clinical manifestations/ Reactivated virus, Detection, Comorbidity, and Treatment from each study.

### Quality assessment

For the quality assessment of the included studies, we used the Joanna Briggs Institute (JBI) Critical Appraisal Checklist for case reports [15] and case series [16]. The eight items in the JBI checklist for case reports cover the patient's demographics, medical history, present clinical state, description of diagnostic tests, therapy, post-intervention clinical state, adverse events, and the providing of takeaways. The JBI checklist for case series is a 10-item scale that assesses the inclusion criteria, method of condition measurement, validity of the diagnostic methods, whether participants were consecutively included, the extent to which participants were included, reporting of the demographic characteristics, clinical information, outcomes, presentation of clinic demographic information, and appropriateness of the statistical analysis [17]. We used the Newcastle–Ottawa Scale (NOS) for assessing the quality of observational cohorts [15]. The scale contains 8 signaling question in 3 different domains (Selection, Comparability, and Outcomes).

### Data Synthesis

We performed a random effect meta-analysis to estimate the proportions of HHV reactivation among patients vaccinated against COVID-19. Since the incidence of reactivation was rare among the included studies, we decided to present the results as events per 1000 observations. Before pooling the effect estimates, we transformed the raw data using the Logit transformation methods to reduce the variation of the study-specific prevalence. I2 test was evaluated to test the heterogeneity among studies. Sensitivity analysis was performed to found the pooled effects in patients who were clinically diagnosed with herpes zoster. All statistical analyses and graphics were carried out using R (version 4.1.3) [18] and the meta package (version 5.5–0) [19]. Furthermore, we describe the results of individual cohorts and case reports/series in a manner of providing a narrative synthesis.

## Results

### Search results

**Fig. 1:**
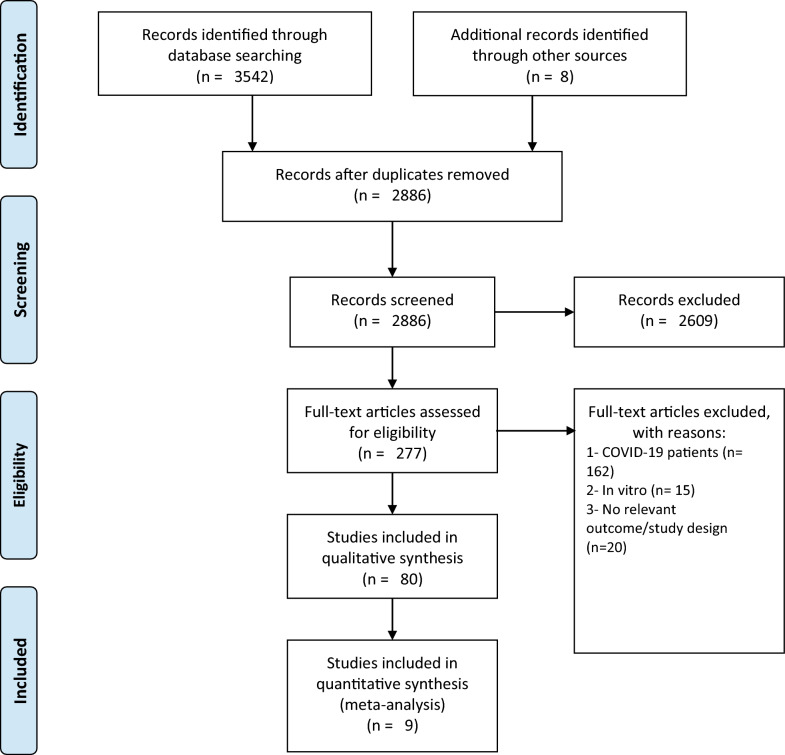
PRISMA flow diagram for article selection

We found a total number of 3542 articles from the mentioned databases. After screening based on the inclusion/exclusion criteria provided, a total number of 80 studies (11 observational cohorts [20–30], 59 case reports [31–88], and 10 case series [89–98]) were eligible for inclusion. It is important to note that most of the studies included in this review were published in 2022 (Fig. 1).

### Qualitative synthesis

#### Evidence from observational cohorts

All included observational studies regarding the possible effect of COVID-19 administration were published in 2022 and 2023. There were 11 studies with this regard mostly focusing on the reactivation of VZV with herpes zoster presentation, showing the paucity of observational studies for other herpesviruses [20–30] (Table 1). Six studies were retrieved their data through registries [20, 23, 25–28]. Other studies were center-based observational cohorts [21, 22, 24]. The sample size of the included cohorts varied from 265 to 2190172. Regarding the type of COVID-19 vaccine administered, 8 studies have evaluated BNT162b2 [21–23, 25–29], 7 have evaluated mRNA-1273 [23–29], 3 have evaluated AZD1222 [21, 24, 26], and one has evaluated CoronaVac [22], Sinopharm (Vero Cell), Sinovac COVID‐19 Vaccine (Vero Cell), Sinopharm/WIBP, CanSinoBio, Zhifei Longcom, KCONECAVAC [30], and Ad26.COV2.S [23]. However, it must be mentioned only 5 studies reported the outcome of interest based on the type of each vaccine [22, 24, 25, 27, 28]. Regarding the type of reactivated HHV, most studies have reported the reactivation of VZV. Only two studies have data regarding the reactivation of HSV [21, 22, 27, 29]. Overall, the results of the included cohorts support the possible association between COVID-19 administration and reactivation of VZV. Five studies have found administrating COVID-19 vaccine is accompanied with higher odds of VZV and HSV reactivation [20, 23, 25, 27, 28]. Among these studies, only Birabaharan, M. reported a non-significant different when comparing with a control group using data from TriNetX database registry (risk ratio: 0.91, 95% CI 0.82–1.01) [20]. Another study by Hertel, M. which used the same database the increased rate of reactivation among the COVID-19 vaccinated group (risk ratio: 1.802, 95% CI 1.680–1.932) [23]. It is noteworthy to mention that the length of their cohort was much longer than Birabaharan, M. (2 years compared with 7 months).

**Table 1:** Characteristics of the included observational studies with their main findings

ID	Author	Year	Country	Type of study (Registry/ Duration)	Population	Total patients	Vaccine	Reactivated virus	Main findings
1	Birabaharan, M	2022	USA	Retrospective cohort (TriNetX/ December 15, 2020 and July 15, 2021)	Patients aged > 18 years who received the mRNA COVID-19 vaccine either as the first or as the second dose	1,306,434	mRNA based	VZV	1- Incidence of VZV reactivation after 28 days of COVID-19 vaccination = 0.1% (1228 of 1,306,434 patients) 2- After matching for baseline variables, there were no significant difference between those who received mRNA COVID-19 vaccine and controls
2	Català, A	2022	Spain	Nationwide, multicenter, cross-sectional observational study (16 February–15 May 2021)	People of any age with any skin reactions within 21 days after any dose of a vaccine	405	BNT162b2, AZD1222	VZV, HSV	1- Among cutaneous reactions, VZV, *n* = 41 (10.1%); and herpes simplex virus (HSV), *n* = 15 (3.7%) were present 2- Varicella-zoster virus reactivation was among the most reported cutaneous reactions
3	Cebeci Kahraman, F	2022	Turkey	Prospective, cross- sectional study (15 April and 15 July 2021)	Patients aged over 18 years, who presented to dermatology or emergency outpatient clinics after having been vaccinated after either the first or second dose	2290	CoronaVac, BioNTech vaccine	VZV, HSV	1- Herpes zoster among those who received the first *n* = 9 (0.4%) and second dose *n* = 10 (0.5%) of CoronaVac; 2- Herpes zoster among those who received the first dose *n* = 8 (4.4%) of BioNTech vaccine; 3- Triggering of herpes simplex [*n* = 90 (4.3%) for CoronaVac and *n* = 9 (4.9%) for BioNTech]
4	Hertel, M	2022	Germany	Retrospective cohort (TriNetX/ 25 November 2021 to 2y backwards	1- Individuals who had received at least one mRNA or adenovirus vector-based COVID-19 vaccine, 2- Those who were not vaccinated against COVID-19	2,190,172	BNT162b2, mRNA-1273, Ad26.COV2.S	VZV	1- 2204 patients developed HZ within 60 days of COVID-19 vaccination; 2- The risk ratio and odds ratio were 1.802 (95% confidence interval [CI] = 1.680; 1.932) and 1.804 (95% CI = 1.682; 1.934) when compared to those not received
5	Lee, T. J	2022	Taiwan	Retrospective cohort (center-based, July 2021 and September 2021)	Patients receiving at least one dose of primary SARS-CoV- 2 vaccine	265	AZD1222, mRNA-1273	VZV	1- Herpes zoster reactivation occurred in 10 patients among mRNA-1273 group versus none in AZD1222 group (6.2% vs 0%, p value = 0.019) 2- Nine patients experienced the first herpes zoster event in their lives
6	Préta, L. H	2022	France	Case/non-case statistical approach (VigiBase up to 30 June 202)	Patients received mRNA COVID-19 vaccines	716 928	BNT162b2 and mRNA-1273	VZV	1- 5931 HZ cases with BNT162b2 and 1797 with mRNA-1273; 2- mRNA COVID-19 vaccines were associated with an increased HZ reporting for BNT162b2 (ROR 2.0, 95% CI 1.8–2.2), mRNA-1273 (ROR 1.5, 95% CI 1.2–1.8) and overall (ROR 1.9, 95% CI 1.8–2.1) compared with those who received influenza vaccine; 3- Reduced risk among younger patients (ROR 0.39, 95% CI 0.36–0.41)
7	Machado, P. M	2022	EULAR Coronavirus Vaccine (COVAX) registry	Cohort from 5 February 2021 to 27 July 2021	Patients vaccinated against SARS-CoV- 2	5121	Pfizer/BioNTech vaccine (70%), 17% AstraZeneca/Oxford and 8% Moderna	VZV	1- 10 HZ cases during the cohort
8	Gringeri, M	2022	U.S. Vaccine Adverse Event Reporting System database	Cohort from 12/13/2020 and 12/03/2021	Patients vaccinated against SARS-CoV- 2	588,323	Pfizer: 548,578,240; Moderna: 361,897,609; Janssen: 33,849,124	VZV, HSV	Out of the 6,195 cases examined in the study, consisting of 5,934 cases of herpes zoster and 273 cases of herpes simplex, more than 90% were classified as non-serious. The analysis revealed a slightly increased likelihood of reporting both herpes zoster (with a relative reporting odds ratio of 1.49) and herpes simplex (with a relative reporting odds ratio of 1.51) infections following vaccination with the Pfizer–BioNTech vaccine. The estimated incidence rates for herpes zoster and herpes simplex were approximately 0.7 per 100,000 and 0.03 per 100,000 cases, respectively
9	Florea, A	2023	Kaiser Permanente Southern California (KPSC)	Cohort from 12/2020–05/2021	Patients vaccinated against SARS-CoV- 2	2,107,823	mRNA-1273 and BNT162b2	VZV	The study cohort consisted of 1,052,362 recipients of the mRNA-1273 vaccine, 1,055,461 recipients of the BNT162b2 vaccine, and 1,020,334 individuals in the comparison group. When compared to individuals who were not vaccinated, the adjusted hazard ratio (aHR) for herpes zoster (HZ) within 90 days after receiving the second dose of the mRNA-1273 vaccine was 1.14 (with a confidence interval of 1.05–1.24), and for the BNT162b2 vaccine, it was 1.12 (with a confidence interval of 1.03–1.22). Among individuals aged 50 years and above who had not received the zoster vaccine, the aHR was also elevated after the second dose of the mRNA-1273 vaccine (1.18 with a confidence interval of 1.06–1.33) and the BNT162b2 vaccine (1.15 with a confidence interval of 1.02–1.29) compared to unvaccinated individuals
10	Fathy, R. A	2022	USA	Cohort as of April 2021	Patients vaccinated against SARS-CoV- 2	588,323	Moderna or the Pfizer–BioNTech	VZV, HSV	Out of 40 reactivated cases, 35 were VZV and 5 were HSV
11	Chen, J	2023	China	Cross‐sectional survey	Autoimmune inflammatory rheumatic diseases vaccinated against SARS-CoV- 2	636	Sinopharm (Vero Cell), Sinovac COVID‐19 Vaccine (Vero Cell), Sinopharm/WIBP, CanSinoBio, Zhifei Longcom, KCONECAVAC	VZV	11 cases with HZ

#### Evidence from case reports/series

There are 149 cases included in this review, which were from 30 different countries around the world. USA (*n* = 21), India (*n* = 15), Greece (*n* = 15), Taiwan (*n* = 9), Saudi Arabia (*n* = 7), Spain (*n* = 7), China (*n* = 6), Switzerland (*n* = 5), Germany (*n* = 4), and Kuwait (*n* = 5) have the most patients. From a 12-year-old adolescent to an elderly patient who was 84 years old, the age range of the patients was varied (Table 2). There were several vaccines that caused reactivation: BNT162b2 mRNA or Pfizer–BioNTech (*n* = 76), Oxford-AstraZeneca (*n* = 22), mRNA-1273 or Moderna (*n* = 17), Sinovac (*n* = 4), BBIBP-CorV or Sinopharm (*n* = 3), Covaxin (*n* = 3), Covishield (*n* = 3), and Johnson and Johnson (*n* = 1). In some of the cases, the exact model of the vaccine was not reported in the paper [53, 90, 99, 100]. Reactivated HHVs included varicella-zoster virus (*n* = 114), cytomegalovirus (*n* = 15), HSV-1 (*n* = 14), Epstein-Barr virus (*n* = 6), and HHV-6 (*n* = 2). The detection methods varied depended on the symptoms of each specific case, but the most common ones were as follows: history and physical examination, clinical symptoms, slit lamp examination, PCR, serum tests, and laboratory evaluation. There were four papers that did not specify the exact method of diagnosing [32, 38, 47, 95]. As a result of the variety of symptoms caused by the reactivation of virus, treatment varied greatly as well. In addition to antiviral drugs (such as acyclovir, valacyclovir, ganciclovir, and valganciclovir), antibiotics, steroids (such as prednisolone), and glucocorticoids (such as dexamethasone) were the most commonly prescribed medicines. The treatment for the patient was not presented in seven studies [32, 59, 72, 74, 75, 97, 101] Many patients reported having comorbidities, of which hypertension, diabetes mellitus, dyslipidemia, chicken pox, and atrial fibrillation were the common ones. There is a detailed description of the specific method of diagnosing and treatment for each case in Additional file 1: Tables S2 and S3.

**Table 2:** Summary baseline characteristic of reported case reports/series

	VZV	HSV	EBV	CMV	HHV-6
Age	55.56 ± 19.70	41.66 ± 20.10	35.20 ± 15.51	60.38 ± 12.38	46.50 ± 9.19
Gender					
Male	53	10	2	8	1
Female	47	4	3	5	1
Comorbidities					
HTN	18	3	0	1	0
Dyslipidemia	8	0	0	0	0
DM	12	0	0	1	0
Heart disease	5	2	1	5	0
Herpetic keratitis	0	2	0	0	0
HIV	1	0	0	1	0
Immune status					
Immunocompetent	98	12	4	3	2
Immunocompromised	2	0	1	10	0
Vaccine type					
Pfizer	54	7	2	8	0
Moderna	7	0	0	3	1
AstraZeneca	20	2	1	1	1
Others	19	5	2	1	0
Vaccine dose					
1st	86	7	5	11	1
2nd	12	2	0	2	1
3rd (Booster)	2	1	0	0	0
Diagnosis					
PCR	34	3	2	13	0
Clinical examination	63	8	3	0	2
Immunoglobulin	2	0	0	0	0
Treatment					
Antiviral	52	2	0	11	0
Pharmacological	5	2	2	0	2
Both	30	10	0	1	0
Clinical manifestation					
Uncomplicated infections	95	14	5	13	2
Serious infections	5	0	0	0	0

#### Results of meta-analysis

**Fig. 2:**
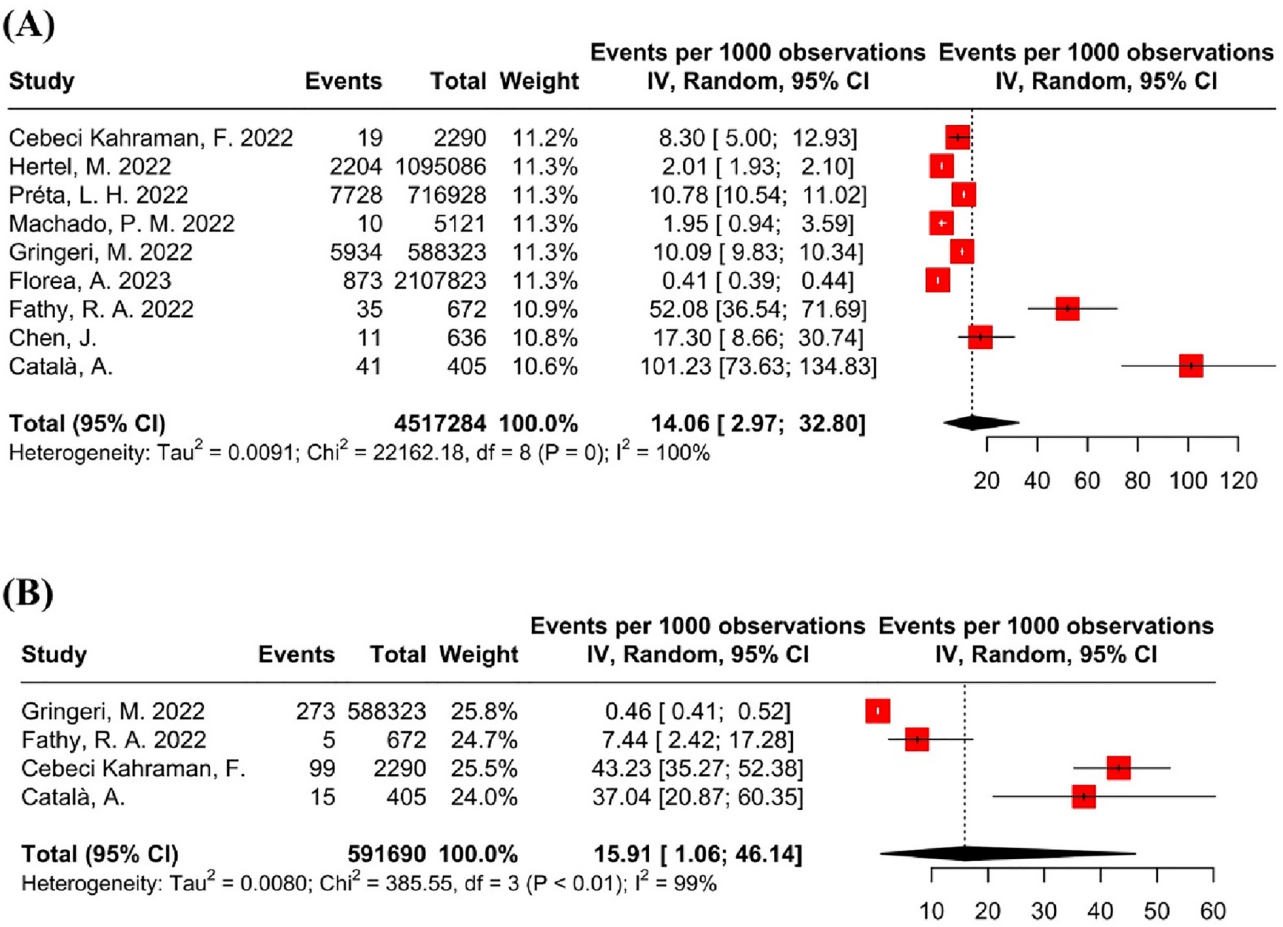
The results of the meta-analysis: **A** varicella-zoster virus. **B** herpes simplex virus

The results of our proportion meta-analysis showed that the rate of VZV reactivation among those who received COVID-19 vaccine was 14 persons per 1000 vaccinations (95% CI 2.97–32.80). Moreover, our meta-analysis for HSV reactivation showed the rate of 16 persons per 1000 vaccinations (95% CI 1.06–46.4) (Fig. 2).

#### Quality assessment

The results of our quality assessment for observational studies showed 6 and 5 studies with low and high risk of bias, respectively. The most domain which differed among the cohorts was regarding providing a comparable group (e.g., control group) which was only present in three studies.

Quality assessment for case reports was performed by JBI checklist, and five studies [33, 40, 49, 61, 78] received an overall score of 8 out of 8, while one study[74] received the lowest score of 4 out of 8, for an overall mean score of 6.2. In terms of scoring, the highest scoring criteria were reporting the demographic characteristics of patients (100%) and the clinical condition of the patients (96%). A precise diagnosis method (49%) and clear reporting of adverse events (55%) received the lowest scores.

Among the case series, one study[96] received a 10/10 score and the lowest score was received by one study (5/10)[89], with an overall mean score of 7.5. Reporting a complete inclusion criteria, demographic information, and clinical information of participants were the highest scoring criteria (10/10, 100%), while the least reported score was for valid methods used for identification of the condition for all participants (4/10, 40%).

The detailed results of quality assessment for all included studies are available in supplementary material.

## Discussion

This study set out with the aim of literature reviewing to examine the potential correlation between the COVID-19 vaccine administration and possible reactivation of the herpesviruses. In our study, 76 reports were included, which comprised patients who had experienced reactivation of different types of herpesviruses after administration of different types of COVID-19 vaccines. The results from observational cohorts showed that the administration of COVID-19 vaccine, especially mRNA-based ones, could be associated with VZV reactivation. It should be noted that most information available was regarding VZV, and not many reports were available for other types of herpesviruses. Few numbers of published records and the nature of observational study would suggest the evidence regarding the association between COVID-19 vaccine and VZV reactivation to be low. Therefore, in addition to the cohorts included for this study, we also reviewed the reported cases of different HHVs reactivation among those who got COVID-19 vaccines. Among different vaccines, BNT162b2 mRNA or Pfizer–BioNTech have been administrated in more than half of the reported cases. Also, among the reactivated HHVs, including VZV, EBV, CMV, HSV-1 and HSV-6, most cases had experienced the reactivation of VZV, which was reported in nearly 70% of case reports, and the less common one was HSV-6 with only 2 cases.

Close to 100% of the adult population is at least once in a lifetime infected by one of the herpes viridea family viruses [102]. This family is known for its ability to indicate latent infection after the primary infection, which can reactivate by external or internal triggers. The latent phase of infection is defined as a situation in which the virus is quiescent, meaning the virus is not replicating which prevents the lytic infection and release of new progeny virus particles; in this mode of infection, external or internal stimuli can reactivate the virus, which defined as switching the latent phase to lytic [103]. Expression of a variety of virus genes during lytic infection leads to make progeny virions. Based on the time of their expression concerning the initial onset of reactivation, they fall into three groups, including IE genes, early (E) genes, and late (L) genes, which encode the proteins whose role in the gene transcription, viral replication, and structural proteins, which result in virion formation and reactivation [103]. There are different sites in which the viruses become latent; VZV mostly stays latent in neurons of dorsal root ganglia, cranial nerve ganglia, and autonomic ganglia, and EBV displays a latent phase in B lymphocytes and epithelial cells. CMV becomes latent in cells of the myeloid and HSV-1 and HSV-2 reactivate from trigeminal ganglia and sacral ganglia, respectively [12, 104–106]. Based on the reactivation of which type of herpes virus family, different kinds of triggers are capable of reactivating the virus. However, on balance, the most typical stimuli are fever, microbial co-infection, tissue injury, stress, immunocompromised situations, hyperthermia, hormonal imbalance, UV light, allogenic stimulation, and cytokines [107].

Vaccine administration can provide some of these triggers, such as hyperthermia and tissue injury as other side effects and also immunodeficiency state; in other words, it may theoretically result in the reactivation of herpes viruses. DNA repair and the immune system are known as the two essential systems for defending against threats; loss of function of DNA repair may lead to disability of production of B and T cells resulting in immunodeficiency [108]. A recent study by Liu et al. involved the pathophysiological alterations after the COVID-19 vaccine in which CD8^+^ T cells reduction, increase in classic monocyte contents, increased NF-κB signaling, and reduced type I interferon responses were reported; they have admitted that in the first 28 days after a vaccine injection, the immune system is in the vulnerable state [109]. Type I IFN receptor signaling in CD8^+^ T cells has an essential role in regulating memory cell response to viral infection and blockage of reactivation [109, 110]. These examples suffice to show that after COVID-19 vaccine administration, reactivation of the herpes virus family may occur. One of the more significant findings to emerge from this study is that, although vaccines are critical for controlling the COVID-19 pandemic, vaccine administration could lead to the reactivation of the herpes virus family. It is true that only few complicated cases have been reported. However, the fact remains that it can influence a large number of people all around the world. Clinical awareness about ways to the early onset diagnosis, preparing the best treatment for patients, and recognizing the patients who are at risk of reactivation are essential.

The results from our study are in line with recent systematic reviews which also reported an association between COVID-19 vaccine and VZV reactivation [111–114]. All previous systematic reviews only included case reports/series regarding the reactivation of VZV. In addition to case reports/series, our systematic review evaluated the available observational evidence regarding VZV reactivation following COVID-19 vaccination, including 6 cohorts. Moreover, our study focused not only on VZV but also on reporting the reported cases available in literature for HSV, EBV, CMV, and HHV-6. Recent systematic review by Martinez-Reviejo eta al. [112] showed most reported cases of VZV reactivation have their symptoms following the first dose of mRNA vaccination and most of the patients were presented with uncomplicated course, with few having serious disease. These results were in line with our findings for HSV, EBV, and CMV.

A number of limitations need to be considered. First, the number of cases that have been reported is inadequate for certainly assessing the correlation between vaccines and HHVs reactivation. Second, these findings are limited by not using the clinical trial design and lack of comparison between vaccinated and non-vaccinated participants. Considerably more work will need to be done to determine the effect of vaccination on HHVs reactivation. On the other hand, our study is the first to review the possible correlation between COVID-19 and HHVs reactivation. The present study provides a comprehensive overview of the published literature and highlights the available data with rigorous quality assessment.

In conclusion, although vaccination has played an essential role in controlling the COVID-19 pandemic, many different side effects should be considered before administration. However, more research on this topic needs to be undertaken before the association between vaccination and reactivation of the herpes virus family is more clearly understood. To date, the reported cases have shown that clinical physicians should be prepared and aware, so they are capable of recognizing their patients who present with the symptoms of herpes virus reactivation after vaccination and providing them with the best prophylaxis and treatment.

### Supplementary Information

**Additional file1: Table S1**. PRISMA 2020 checklist. **Table S2.** Databases searched and search strategies employed. **Table S3.** Detailed results of included case reports. **Table S4.** Detailed results of included case series. **Table S5.** The results of quality assessment for observational studies. **Table S6.** The results of quality assessment for case reports. T**able S7.** The results of quality assessment for case series.

## Data Availability

All relevant data are within the manuscript and its Additional file 1.
