# Supplementary material for: Herpesviruses reactivation following COVID-19 vaccination: a systematic review and meta-analysis
Source: Eur J Med Res. 2023 Aug 10;28:278. doi: 10.1186/s40001-023-01238-9 (PMC10413536; doi:10.1186/s40001-023-01238-9)
Supplement: Supplementary file 1 — Additional file1: Table S1. PRISMA 2020 checklist. Table S2. Databases searched and search strategies employed. Table S3. Detailed results of included case reports. Table S4. Detailed results of included case series. Table S5. The results of quality assessment for observational studies. Table S6. The results of quality assessment for case reports. Table S7. The results of quality assessment for case series. [file 40001_2023_1238_MOESM1_ESM.docx]

**Supporting Information**

**Herpesviridae reactivation following COVID-19 vaccination: Evidence from a systematic review.**

**Table S1.** PRISMA 2020 checklist

**Table S2.** Databases searched and search strategies employed**.**

**Table S3.** Detailed results of included case reports.

**Table S4.** Detailed results of included case series.

**Table S5.** The results of quality assessment for observational studies.

**Table S6.** The results of quality assessment for case reports.

**Table S7.** The results of quality assessment for case series.

**Table S1.** PRISMA 2020 checklist

| **Section and Topic** | **Item #** | **Checklist item** | **Location where item is reported** |
| --- | --- | --- | --- |
| **TITLE** | | |  |
| Title | 1 | Identify the report as a systematic review. | 1 |
| **ABSTRACT** | | |  |
| Abstract | 2 | See the PRISMA 2020 for Abstracts checklist. | 2 |
| **INTRODUCTION** | | |  |
| Rationale | 3 | Describe the rationale for the review in the context of existing knowledge. | 4-5 |
| Objectives | 4 | Provide an explicit statement of the objective(s) or question(s) the review addresses. | 4-5 |
| **METHODS** | | |  |
| Eligibility criteria | 5 | Specify the inclusion and exclusion criteria for the review and how studies were grouped for the syntheses. | 6 |
| Information sources | 6 | Specify all databases, registers, websites, organisations, reference lists and other sources searched or consulted to identify studies. Specify the date when each source was last searched or consulted. | 6 |
| Search strategy | 7 | Present the full search strategies for all databases, registers and websites, including any filters and limits used. | 6, Table S1 |
| Selection process | 8 | Specify the methods used to decide whether a study met the inclusion criteria of the review, including how many reviewers screened each record and each report retrieved, whether they worked independently, and if applicable, details of automation tools used in the process. | 6 |
| Data collection process | 9 | Specify the methods used to collect data from reports, including how many reviewers collected data from each report, whether they worked independently, any processes for obtaining or confirming data from study investigators, and if applicable, details of automation tools used in the process. | 6-7 |
| Data items | 10a | List and define all outcomes for which data were sought. Specify whether all results that were compatible with each outcome domain in each study were sought (e.g. for all measures, time points, analyses), and if not, the methods used to decide which results to collect. | 6-7 |
|  | 10b | List and define all other variables for which data were sought (e.g. participant and intervention characteristics, funding sources). Describe any assumptions made about any missing or unclear information. | 6-7 |
| Study risk of bias assessment | 11 | Specify the methods used to assess risk of bias in the included studies, including details of the tool(s) used, how many reviewers assessed each study and whether they worked independently, and if applicable, details of automation tools used in the process. | 7-8 |
| Effect measures | 12 | Specify for each outcome the effect measure(s) (e.g. risk ratio, mean difference) used in the synthesis or presentation of results. | 7-8 |
| Synthesis methods | 13a | Describe the processes used to decide which studies were eligible for each synthesis (e.g. tabulating the study intervention characteristics and comparing against the planned groups for each synthesis (item #5)). | 7-8 |
|  | 13b | Describe any methods required to prepare the data for presentation or synthesis, such as handling of missing summary statistics, or data conversions. | 7-8 |
|  | 13c | Describe any methods used to tabulate or visually display results of individual studies and syntheses. | 7-8 |
|  | 13d | Describe any methods used to synthesize results and provide a rationale for the choice(s). If meta-analysis was performed, describe the model(s), method(s) to identify the presence and extent of statistical heterogeneity, and software package(s) used. | 7-8 |
|  | 13e | Describe any methods used to explore possible causes of heterogeneity among study results (e.g. subgroup analysis, meta-regression). | 7-8 |
|  | 13f | Describe any sensitivity analyses conducted to assess robustness of the synthesized results. | 7-8 |
| Reporting bias assessment | 14 | Describe any methods used to assess risk of bias due to missing results in a synthesis (arising from reporting biases). | 7-8 |
| Certainty assessment | 15 | Describe any methods used to assess certainty (or confidence) in the body of evidence for an outcome. | 7 |
| **RESULTS** | | |  |
| Study selection | 16a | Describe the results of the search and selection process, from the number of records identified in the search to the number of studies included in the review, ideally using a flow diagram. | 9 |
|  | 16b | Cite studies that might appear to meet the inclusion criteria, but which were excluded, and explain why they were excluded. | 9 |
| Study characteristics | 17 | Cite each included study and present its characteristics. | 9 |
| Risk of bias in studies | 18 | Present assessments of risk of bias for each included study. | 15 |
| Results of individual studies | 19 | For all outcomes, present, for each study: (a) summary statistics for each group (where appropriate) and (b) an effect estimate and its precision (e.g. confidence/credible interval), ideally using structured tables or plots. | Table 1 |
| Results of syntheses | 20a | For each synthesis, briefly summarise the characteristics and risk of bias among contributing studies. | 14-15 |
|  | 20b | Present results of all statistical syntheses conducted. If meta-analysis was done, present for each the summary estimate and its precision (e.g. confidence/credible interval) and measures of statistical heterogeneity. If comparing groups, describe the direction of the effect. | 14-15 |
|  | 20c | Present results of all investigations of possible causes of heterogeneity among study results. | 14-15 |
|  | 20d | Present results of all sensitivity analyses conducted to assess the robustness of the synthesized results. | 14-15 |
| Reporting biases | 21 | Present assessments of risk of bias due to missing results (arising from reporting biases) for each synthesis assessed. | 15 |
| Certainty of evidence | 22 | Present assessments of certainty (or confidence) in the body of evidence for each outcome assessed. | 15 |
| **DISCUSSION** | | |  |
| Discussion | 23a | Provide a general interpretation of the results in the context of other evidence. | 16 |
|  | 23b | Discuss any limitations of the evidence included in the review. | 19 |
|  | 23c | Discuss any limitations of the review processes used. | 19 |
|  | 23d | Discuss implications of the results for practice, policy, and future research. | 17-18 |
| **OTHER INFORMATION** | | |  |
| Registration and protocol | 24a | Provide registration information for the review, including register name and registration number, or state that the review was not registered. |  |
|  | 24b | Indicate where the review protocol can be accessed, or state that a protocol was not prepared. |  |
|  | 24c | Describe and explain any amendments to information provided at registration or in the protocol. |  |
| Support | 25 | Describe sources of financial or non-financial support for the review, and the role of the funders or sponsors in the review. |  |
| Competing interests | 26 | Declare any competing interests of review authors. |  |
| Availability of data, code and other materials | 27 | Report which of the following are publicly available and where they can be found: template data collection forms; data extracted from included studies; data used for all analyses; analytic code; any other materials used in the review. |  |

*From:*  Page MJ, McKenzie JE, Bossuyt PM, Boutron I, Hoffmann TC, Mulrow CD, et al. The PRISMA 2020 statement: an updated guideline for reporting systematic reviews. BMJ 2021;372:n71. doi: 10.1136/bmj.n71

For more information, visit: <http://www.prisma-statement.org/>

**Table S2. Databases searched and search strategies employed**

**Date: 10/25/2022- Total: 3260, Pubmed:970, EMBASE: 1294, WOS: 996, After dup: 2671**

| **Database** | **Search strategy** | **Result** |
| --- | --- | --- |
| PubMed | (((((("COVID-19"[Mesh]) OR "SARS-CoV-2"[Mesh]) OR (COVID-19[Title/Abstract])) OR (Coronavirus[Title/Abstract])) OR (nCoV[Title/Abstract])) OR (SARS-Cov-2[Title/Abstract])) AND ((((((((((((((((((((((("Herpesviridae"[Mesh]) ) OR (HHV‑1[Title/Abstract])) OR (HSV-1[Title/Abstract])) OR (Herpes simplex virus-1[Title/Abstract])) OR (HHV-2[Title/Abstract])) OR (HSV-2[Title/Abstract])) OR (Herpes simplex virus-2[Title/Abstract])) OR (HHV-3[Title/Abstract])) OR (Varicella zoster virus[Title/Abstract])) OR (VZV[Title/Abstract])) OR (HHV-4[Title/Abstract])) OR (Epstein–Barr virus[Title/Abstract])) OR (EBV[Title/Abstract])) OR (HHV-5[Title/Abstract])) OR (Cytomegalovirus[Title/Abstract])) OR (CMV[Title/Abstract])) OR (HHV-6A[Title/Abstract])) OR (HHV-6B[Title/Abstract])) OR (Roseolovirus[Title/Abstract])) OR (HHV-8[Title/Abstract])) OR (Kaposi's sarcoma-associated herpesvirus[Title/Abstract])) OR (KSHV[Title/Abstract])) | **970** |
| Web of Science | **TS=("HHV 1" OR "HSV-1" OR "Herpes simplex virus-1" OR "HHV-2" OR "HSV-2" OR "Herpes simplex virus-2" OR "HHV-3" OR "Varicella zoster virus" OR "VZV" OR "HHV-4" OR "Epstein–Barr virus" OR "EBV" OR "HHV-5" OR "Cytomegalovirus" OR "CMV" OR "HHV-6A" OR "HHV-6B" OR "Roseolovirus" OR "HHV-8" OR "Kaposi's sarcoma-associated herpesvirus" OR "KSHV")**  **AND**  **TS=(”COVID-19” OR ”Coronavirus” OR ”nCoV” OR ”SARS-Cov-2”)** | **996** |
| EMBASE | ((HHV‑1):ab,ti OR ((HSV-1):ab,ti) OR (('Herpes simplex virus-1'):ab,ti) OR ((HHV-2):ab,ti) OR ((HSV-2):ab,ti) OR (('Herpes simplex virus-2'):ab,ti) OR (('Herpes simplex virus'):ab,ti) OR ((HHV-3):ab,ti) OR (('Varicella zoster virus'):ab,ti) OR ((VZV):ab,ti) OR ((HHV-4):ab,ti) OR (('Epstein Barr virus'):ab,ti) OR ((EBV):ab,ti) OR ((HHV-5):ab,ti) OR ((Cytomegalovirus):ab,ti) OR ((CMV):ab,ti) OR ((HHV-6A):ab,ti) OR ((HHV-6B):ab,ti) OR ((Roseolovirus):ab,ti) OR ((HHV-8):ab,ti) OR (('Human herpesvirus 8'):ab,ti) OR (('Kaposis sarcoma-associated herpesvirus'):ab,ti) OR ((KSHV):ab,ti)  AND  ('coronavirus disease 2019':ab,ti OR 'covid 19':ab,ti OR coronavirus:ab,ti OR ncov:ab,ti OR 'sars cov 2':ab,ti) | **1294** |

**S3 Table:** Detailed results of included case reports.

| **Author** | **Year** | **Country** | **Total Patients (n)** | **Age** | **Vaccine** | **Clinical manifestations/ Reactivated virus** | **Detection** | **Comorbidity** | **Treatment** |
| --- | --- | --- | --- | --- | --- | --- | --- | --- | --- |
| Abu-Rumeileh, S. [1] | 2022 | Germany | 3 | 1. 82 2. 70 3. 63 | 1. BNT162b2 2. ChAdOx1 3. ChAdOx1 | 1. VZV meningitis with Ramsay-Hunt syndrome sine herpete 2. VZV meningitis sine herpete 3. VZV meningoradiculitis sine herpete | PCR/ IgG AND IgA | 1. Coronaropathy, hypertension, dyslipidemia, obesity 2.none 3 .none | acyclovir |
| Al-Dwairi, R. A. [2] | 2022 | Jordan | 1 | 50 | Pfizer/BioNTech | left eye redness, tearing and pain in the left eye/ HSV-1 | clinical exam | hypertension and ischemic heart disease and herpetic keratitis | Acyclovir, prednisolone, fluorometholone, moxifloxacin |
| Algaadi [3] | 2021 | Saudi Arabia | 1 | 65 | Chimpanzee adenovirus vectored vaccine | Painful vesicles and burning sensation right aspect of chest / VZV | History and physical examination | Coronary heart disease, hypertension, and diabetes mellitus | Acyclovir and topical fusidic acid |
| Alkhalifah [4] | 2021 | Saudi Arabia | 2 | 1. 42   2. 29 | 1. Pfizer/BioNTech (BNT162B2) 2.Pfizer/BioNTech (BNT162B2) | 1. Drop in vision, ocular pain. Photophobia, and periorbital headache 2. Redness, pain, photophobia, and blurring of vision of the left eye / HSV-1 | 1. Slit lamp examination, corneal scraping, and PCR 2. Slit lamp examination, and PCR | 1. Recurrent herpetic kerato-uveitis of the right eye, previous attack of necrotizing stromal keratitis, corneal scarring and thinning 2. Recurrent herpetic endotheliitis with and old overlying stromal scarring of the left eye | 1. Acyclovir oral, broad spectrum fortified antibiotics 2. Acyclovir oral, acyclovir ointment, topical streoids, and prophylactic topical moxifloxacin |
| Ardalan [5] | 2021 | Iran | 1 | 28 | Oxford-AstraZeneca | Herpes-like lesions on the right upper lid, burning sensation, and painful skin rashes, and blisters on the right upper eyelid, followed by upper eyelid edema / HSV | History and clinical symptoms | HSV infection following a trauma to the right eye with an object in childhood causing periods of cold sore | Topical antibiotic therapy including sulfacetamide eye drop and ketotifen eye drop, vitamin A ointment |
| Arora [6] | 2021 | India | 1 | 60 | COVAXIN | Fluid-filled lesions over the thigh / VZV | Physical examination | Type II diabetes mellitus and hypertension | Oral valacyclovir along with topical fusidic acid for local application |
| Atiyat [7] | 2021 | USA | 1 | 36 | Pfizer | Unilateral ventral portion of right arm and right anterior chest painful rash / VZV | Physical examination | Shingles, HIV | Acyclovir and cefazolin, gabapentine, Biktarvy (bictegravir/emtricitabine/tenofovir) |
| Aksu [8] | 2021 | Turkey | 1 | 68 | NA | Multiple pinheaded vesicular lesions upon an erythematous  base occupying an area on his right mammary region and  back corresponding to T3–T5 dermatomes | Physical examination | Hypertension, dysrhythmia and anxiety | Valaciclovir 1 g thrice daily for 1 week, acyclovir cream, and paracetamol  for pain. |
| Buranasakda [9] | 2022 | Thailand | 2 | 1. 34 2. 32 | 1. Sinovac 2. Oxford-AstraZeneca | 1. Vesicular lesion on the left side of the waist, headache, and vomiting, stiff neck 2. Bilateral temporal headache with fever / VZV | 1. Physical examination and lab tests, PCR 2.physical examination and lab tests, PCR | 1. Asthma, varicella twice at a young age 2. Asthma and chronic hepatitis B with seroconversion | 1. Intravenous acyclovir 2. Intravenous acyclovir |
| Chiu [10] | 2021 | Taiwan | 3 | 1. 71 2. 46 3. 42 | 1. Moderna 2. Oxford-AstraZeneca 3. Oxford-AstraZeneca | 1. Grouped erythematous papules and vesicles on the left flank with itching and pain 2. and 3. Pain and itch over ipsilateral flank / VZV | 1. Physical examination 2. and 3. Physical examination | 1. None 2. NA 3. NA | 1. Oral acyclovir 2. and 3. Oral acyclovir |
| Daouk, S. K. [11] | 2022 | USA | 1 | 12 | BNT162b2 | Rash with zoster meningitis/ VZV | PCR | healthy | N.A |
| Dermawan, A. [12] | 2022 | Australia | 1 | 84 | AstraZeneca | gradually worsening right upper limb weakness with an associated painful rash/ VZV | Physical examination, blood test and PCR | type 2 diabetes, hypertension, hypercholesterolaemia and shingles in the right ophthalmic nerve (V1) dermatome | oral valacilovir |
| Eid [13] | 2021 | Lebanon | 1 | 79 | Moderna | Itchy and tender lesions over the right thigh / VZV | Physical and dermatologic examination | Hypertension, coronary artery disease, and antineutrophilic cytoplasmic antibody-related glomerulonephritis | Systemic antiviral treatment |
| Fukuoka [14] | 2021 | Japan and USA | 5 | 1. 63 2. 70 3. 84 4. 97 5. 59 | BNT162b2 mRNA | Unilateral acute rash for less than seven days with or without pain. Case 2 also had a skin reaction on her right orbit and ear / VZV | Physical examination | 1. Asthma 2. Hypertension 3. Arrythmia, stroke, Meniere's disease 4. Hypertension, stroke, dementia 5. None | None, except case 2, who was treated with an antiviral drug |
| Girardin, F. R. [15] | 2022 | Switzerland | 1 | 44 | mRNA-1273 SARS-CoV-2 vaccine | acute fatigue, palpable spleen and tonsillitis with white spots/ EBV | Examination, serology, ultrasonography | none | NA |
| Herzum, A. [16] | 2022 | Italy | 1 | 24 | BioNTech/Pfizer | aint maculopapular rash, scaly, erythematous, and non-itchy on his limbs and trunk/ EBV | Examination, complete blood cell count, liver, and kidney function and Antibody test | none | NA |
| Jeong [17] | 2021 | Korea | 1 | 54 | Pfizer-BioNTech | A decrease in the vestibulo-occular reflex gain without catch-up saccade in the left lateral semicircular canal / HSV-1 | Video head impulse test | Hypertension | Five days of hospitilization with vestibular suppressants |
| Jiang, Z. H. [18] | 2022 | Taiwan | 3 | 1.79 , 2.91 . 3.25 | Oxford-AstraZeneca | Dermatome, fever, HZV | examination, PCR | 1.Late-onset atopic dermatitis Parkinsonism, 2.Chronic kidney disease, 3.none | NA |
| Kerr [19] | 2022 | Ireland | 1 | 39 | BNT162b2 Comirnaty (Pfizer BioNTech) | Vesicular torso rash, occipital headache associated with nausea, vomitting, and fever / VZV | PCR, Physical examination, routine blood work, and lumbar puncture | Chicken pox in childhood | Intravenous aciclovir |
| Kluger [20] | 2022 | Finland | 4 | 1. 39 2. 70 3. 63 4. 51 | 1. BNT162b2 mRNA vaccine 2. BNT162b2 mRNA vaccine 3. BNT162b2 mRNA vaccine 4. Oxford-AstraZeneca | 1. Acute facial rash, a few sparse lesions, puffiness and dusy red periorbital edema, palpabale cervical lymph nodes and sensitive preauricular lymph node enlargement 2. Typical HZ eruption 3. Typical HZ eruption 4. HZ of the scalp, followed by pain in the face and possibly in the oral cavity / HSV-1 and VZV | 1. Physical examination 2. Phsyical examination and PCR 3. Physical examination 4. Physical examination | 1. Psoriasis 2. Myeloma 3. Childhood chicken pox and rheumatoid arthritis  4. Relapsing palatal herpes | 1. Valaciclovir 2.Valaciclovir 3. None 4. NA |
| Koh [21] | 2022 | Korea | 1 | 24 | Pfizer BNT162b2 | Headache, myalgia, and fatigue, small vesicles on the left upper arm / VZV | Physical examination, lab tests, and CSF PCR | Varicella in childhood and aseptic meningitis at 12 years of age | Oral famciclovir |
| Li [22] | 2021 | China | 2 | 1. 60 2. 51 | 1. Sinovac 2. Sinovac | 1. Tearing associated with redness, photophobia and worsened vision in the right eye / HSV-1 2. Redness and blurry vision in the left eye / VZV | 1. Slit lamp examination 2. Slit lamp examination and PCR | 1. Penetrating eratoplsty (PKP) in the same eye one year ago for corneal scarring caused by herpes simplex keratitis (HSK) 2. None | 1. Topical ganciclovir 2. Topical steroids, topical and oral ganciclovir |
| Lim [23] | 2022 | Singapore | 2 | 1. 78 2. 80 | 1. Pfizer-BioNTech mRNA 2. Pfizer-BioNTech mRNA | 1. Throat pain, difficulty swallowing, and an aching sensation on the left mastoid process and left temporal scalp /N.A 2. Multiple tense vesicles and bullae on an erythematous base along the left L5 dermatome, in addition to scattered vesicles and erosions on the bilateral posterior aspect of the auricular regions and on the right side of the neck/vzv / VZV | 1. Physical examination and otolaryngologic endoscopy 2. Physical examination, Tzanck smear, and PCR | 1. Ischemic heart disease, hypertension, and glaucoma 2. Hypertnesion, diabetes mellitus, obesity, left-breast cancer in remission,and well-controlled pemphigoid | 1. Valacyclovir 2. Intravenous acyclovir |
| Lin, T. Y. [24] | 2022 | Taiwan | 1 | 14 | BNT162b2 vaccination | drowsy consciousness, mottling skin, jaundice, respiratory distress, and hypotension, EBV | examination, blood test, liver function, PCR, Abbott RealTime EBV assay | none | intravenous immunoglobulin, methylprednisolone, prednisolone |
| Lo [25] | 2022 | Australia | 1 | 42 | Pfizer -BioNTech mRNA | Left ARN / VZV | Aqueous and vitreous tap PCR | NA | Combinnation therapy, including intravitreal foscarnet, oral valaciclovir and prednisolone, topical dexamethasone and atropine, and barrier retinal laser |
| Lv, Y. [26] | 2022 | China | 1 | 58 | Sinopharm, Sinovac | perianal discomfort and abdominal distention, CMV | ESR, CRP, stool examination, PCR, colonoscopy | none | ganciclovir intravenously and oral |
| Maldonado [27] | 2021 | Spain | 2 | 1. 79 2. 56 | 1. Pfizer-BNT162b2 2. Pfizer-BNT162b2 | 1. Elevated erythematous lesions with vesicles on the right-hand-side lumbar area spreading to the lower back, hip, groin, and right-hand-side front and inner thigh 2. Fever, with haemorrhagic vesicles upon and rythematous base, chest pain, and pain in the arm on the same side / VZV | 1. Physical examination 2. Physical examination | 1. Hypercholesterolemia, hyperuricemia, hypertension, implanted pacemaker 2. Hypertension, COVID-19 | 1. Aciclovir (oral and topical), non-steroidal anti-inflammatory drugs 2. Gabapentine, oral vitamin B complex |
| Maranini [28] | 2021 | Italy | 1 | 41 | BNT162b2 mRNA | Itchy and tender skin rash over the volar surface of the right forearm / VZV | Dermatological examination and serological tests | HLA_B27 related ankylosing spondylitis, migraine and an annually monitored microprolactinoma | Systemic antiviral treatment with acyclovir |
| Maruki [29] | 2021 | Japan | 1 | 71 | BNT162b2 mRNA vaccine | Fever and headache / VZV | Physical examination and CSF examination and PCR | Immunoglobulin A (IgA) nephritis, chicken pox in childhood | Intravenous acyclovir |
| Medhat, R. [30] | 2022 | UAE | 4 | 46 | BNT162B12 Mrna | long severe headache/ VZV | pcr | dyslipidemia | acyclovir |
| Mishra [31] | 2021 | India | 1 | 71 | COVID-19 vaccine | Reduced vision associated with redness and pain in the right eye / VZV | Eye examination and PCR | NA | NA |
| Muhie [32] | 2021 | Ethiopia | 1 | 72 | COVAXIN | Left lateral chest pain, rash on the left lateral chest, gradual pain / VZV | Physical examination | None | Analgesics |
| Munasinghe [33] | 2022 | Sri Lanka | 1 | 71 | BNT162b2 mRNA vaccine | Crops of pustular vesicles of differing age in potero-lateral aspects of left arm spreading to the anterior forearm in a multidermatomal pattern / VZV | Physical exmination and PCR | Childhood history of chicken pox, diabetes and hypertension, moderately differentiated adenocarcunoma of rectum, past myocardial infarction with dual vessel disease | Oral acyclovir, antibiotics, topical soframycin cream and pottasium permanganate compression dressings |
| Nastro [34] | 2021 | Italy | 2 | 84 | Pfizer-BioNTech | A burning pain on the distal part of the right leg and foot, followed by multiple non-confluent purpuric papules and vesicles n the same sites / VZV | Serum tests, Punch biopsy, PCR | Chronic kidney disease, depressive disorder | Oral famciclovir |
| Nishimoto [35] | 2022 | Japan | 1 | 72 | Pfizer-BNT 162b2 | Serious, intolerable stomachache and back pain, raised bumps / VZV | PCR | acute myelomonocytic leukemia ,Type 2 diabetes mellitus, Paroxysmal atrial fibrilation, Pnuemocystis pneumonia | Intravenous acyclovir |
| Ota [36] | 2022 | Japan | 1 | 74 | Pfizer-BioNTech | Mild vesicular rash on the left part of the chest / VZV | Immunoglobulin serum tests | Rectal cancer | Oral valacyclovir |
| Ozdemir [37] | 2021 | Turkey | 2 | 1. 23 2. 21 | Inactivated COVID-19 vaccine | 1. Itchy and painful rash on the lower back and pain 2. Painful blisters at the abdomen / VZV | 1. Physical examination 2. Physical examination | 1. healthy 2. None | 1. Brivudine 2. Valacyclovir |
| Palanivel [38] | 2021 | India | 2 | 1. 25 2. 55 | Oxford-AstraZeneca | 1. Vesicles on the back of the right thigh 2. Painful, crusty, hemorrhagic rashes on the left upper thigh and upper buttock region / VZV | 1. Dermatological examination 2. Dermatological examination | 1. NA 2. NA | 1. Acyclovir topical cream and oral valaciclovir 2. Topical acyclovir and a combination of vitamin B12 and pregabalin |
| Papasavvas [39] | 2021 | Switzerland | 3 | 1. 73 2. 69 3. 72 | 1. Pfizer BioNTech 2. Pfizer BioNTech 3. Moderna mRNA vaccine | 1. HZO in the right V1 dermatome 2. HZO in the V1 left dermatome 3. Eruption in the left V1 dermatome / VZV | 1. Physical examination 2. Physical examination 3. Physical examination and slit lamp examination | 1. Ocular sarcoidosis 2. Trauma to the right eye 3. Cataract | 1. Valacyclovir and capsaicin ointment 2. Oral valacyclovir, acyclovir ointment, Paracetamol, and Pregabalin 3. Valacyclovir, acyclovir ointment, a combined antibiotic, and dexamethasone |
| Pedrazini [40] | 2021 | Brazil | 1 | 53 | Oxford-AstraZeneca vaccine | Fever, joint pain, chills, headache, and a reddish, erythematous lesion with mild ithching on the posterior right thigh, above the knee joint / PR | Dermatological examination | Hashimoto's Thyroiditis | L-Lysine capsules, and restriction of foods and supplements containing the amino acid L-Arginine |
| Plub [41] | 2021 | Germany | 1 | 67 | Oxford-AstraZeneca | Fever, weakness and arthralgia of the knees, hips and shoulders, mediastinal lymphadenopathy and hepatic steatosis / CMV | Serology tests and PCR | Atrial fibrilation, hypertension, obesity, and degenerative knee joint disease | Oral valganciclovir |
| Poudel, S. [42] | 2022 | USA /Nepal | 1 | 17 | Moderna | Bell’s pulsy / VZV,HSV1,HSV6 | Cranial nerve examination | N.A | prednisolon/steroid/ acyclovir |
| Ryu [43] | 2022 | Korea | 1 | 87 | BNT162b2 mRNA | Sudden visual impairement / VZV | NA | NA | Valacyclovir and topical corticosteroids |
| Said [44] | 2021 | USA | 2 | 1. 58 2. 70 | 1. BNT162b2 2. mRNA-1273 (Moderna) | 1. New-onset diffuse eruption, fatigue, and chills, and diarrhea 2. Diffuse erythematous vesicular rash, and a pruritic skin eruption with associated daily fevers / VZV | 1. Dermatological examination, Laboratory evaluation, PCR, and immunohistochemistry 2. Dermatological examination, and PCR | 1. Acute myeloid leukemia 2. Giant cell arteritis and resolved SARS-CoV-2 infection | 1. Oral valacyclovir 2. Oral valacyclovir, and intravenous acyclovir |
| Sangoram, R. [45] | 2022 | India | 1 | 40 | COVISHIELD-chAdox1s recombinant | photophobia,anterior uvetitis/ HSV1 | PCR | ? | antiviral therapy/ topical steroids/cycloplegic agent |
| Santovito [46] | 2021 | Switzerland | 1 | 27 | Pfizer-BioNTech | Itchy, pink, papular rashes / VZV | Dermatological examination | Chickenpox during childhood | Prednisone, hydroxyzine, and mupirocin ointment |
| Singh, J. [47] | 2022 | India | 1 | 29 | Covishield | photophobia and Retinitis/HSV | PCR | ? | antiviral and steroid |
| Tang, L. [48] | 2021 | China | 1 | 43 | Inactivated SARS-CoV-2 vaccine | Malaise, vomitting, and fever (HLH)/ EBV | Laboratory and imaging tests (EBV DNA+) | None | Dexamethasone acetate |
| Tang, W. R. [49] | 2022 | Taiwan | 1 | 51 | ChAd0x1 nCoV-19 | Fever and generalized soreness / EBV | Contrast-enhanced computed tomography of the chest, pathology examination | Orthotopic heart transplantation for idiopathic dilated cardiomyopathy, hemodialysis for end-stage renal disease, and immunosuppressants | 50% reuction in immunosuppressant dosages in conjuction with rituximab |
| Tanizaki, R. [50] | 2022 | Japan | 1 | 60 | BNT162b2 mRNA COVID-19 | Fever, headache, sore throat, and lumbar pain / VZV | ELISA | Type-2 diabetes mellitus | Oral acetaminophen, carbamazepine, amitriptyline |
| Thimmanagari [51] | 2021 | USA | 2 | 1. 42 2. 49 | 1. Johnson and Johnson COVID-19 vaccine 2. Moderna vaccine | 1. Worsening painful lesions on the left side of the forehead, blurred vision, and watery discharge from the eyes 2. Worsening burning pain on the right side of the forehead and blurred vision / VZV | 1. Physical examination and lab tests 2. Physical examination and lab tests | 1. Asthma, chickenpox in childhood 2. Bipolar disorder, schizophrenia, and hyperlipidemia | 1. Systemic antiviral and antiviral eye drops 2. Systemic antiviral therapy |
| Thonginnetra, S. [52] | 2022 | Thailand | 1 | 16 | BBIBP-CorV | painful skin eruption/ herpes zoster | N.A | None | acyclovir |
| Tripathy, D. M. [53] | 2022 | India | 2 | 1. 54 2. 52 | 1. Covishield 2. Covaxin | 1. Multiple grouped vesicles and bullae with severe burning and hyperalgesia 2. Multiple asymptomatic erythematous to violaceous raised lesions / VZV | 1. Physical examination 2. Physical examination, histopathology | 1. NA 2. None | 1. Parenteral acyclovir and broad-spectrum antibiotics 2. Lesion directed therapy |
| Vallianou [54] | 2022 | Greece and China | 1 | 53 | BNT162b2 mRNA | Painful erythematous rash on the right upper back / VZV | Physical examination | None | Valacyclovir |
| Van Dam [55] | 2021 | Netherlands | 2 | 1. 29 2. 34 | 1. Tozinameran 2. Tozinameran | 1. Painful grouped vesicles on the left lateral of the ox coccygas 2. Painful and swollen inguinal lymph nodes with a rash on the right leg / VZV | 1. Physical examination 2. Physical examination and PCR | 1. Chickenpox three times as a child 2. Ulceritive colitis, chickenpox as a child | 1. None 2. Valacyclovir |
| Wang, C. S. [56] | 2022 | Taiwan | 1 | 40 | COVID-19 mRNA-1273 vaccine | Severe itching skin lesions / HHV-6 | Cutaneous examination, histopathological exam | Urticaria several months ago | Oral prednisolone |
| You, I. C. [57] | 2022 | Korea | 1 | 74 | BNT162b2 | Severe headache and forehead pain, eyelid swelling, and photophobia / VZV | Occular examination and slit lamp examination | Hypertension, diabetes mellitus, and left hemiplegia, chicken pox in childhood | Intravenous acyclovir and topical acyclovir ointment and levofloxacin 1.5% eye drop |
| Zhang, L. W. [58] | 2022 | China | 1 | 65 | CoronaVac | Multiple painful vesicles and erythema / HZ | clinical exam and pathology | N.A | valacyclovir, gabapentin, vitamin B1 and mecobalamine |
| Zheng [59] | 2021 | USA | 1 | 62 | Pfizer-BioNTech | Eye redness, decreased vision, and floaters/retinal necrosis / VZV | Still lamp examination, funduscopic examination, and PCR | NA | Intravitreal and intravenous antiviral therapy |

**Table S4.** Detailed results of included case series.

| **Author** | **Year** | **Country** | **Total Patients (n)** | **Age** | **Vaccine** | **Clinical manifestations/ Reactivated virus** | **Detection** | **Comorbidity** | **Treatment** |
| --- | --- | --- | --- | --- | --- | --- | --- | --- | --- |
| Alkwikbi [60] | 2022 | Saudi Arabia | 4 | 1. 18 2. 40 3. 32 4. 29 | 1. Pfizer-BioNTech 2. Pfizer-BioNTech 3. Oxford-AstraZeneca 4. Pfizer-BioNTech | 1. Pain, photophobia, and lacrimation from the right eye 2. Pain, tearing, and photophobia in the right eye 3. Pain, redness, and blurry vision in the right eye 4. Pain, tearing, and redness in the left eye / HSV-1 | 1. Fluorescein staining 2. Fluorescin staining 3. Slit lamp examination 4. Slit lamp examination | 1. Swimming with contact lenses 2.None 3. None 4. None | 1. Ganciclovir ophthalmic gel eye drops and lubricants 2. Lubrication, ganciclovir ophthalmic gel,a dn oral acyclovir 3. Prednisone with gradual tapering, and cyclopentolate 4. Ganciclovir ophthalmic gel, and oral acyclovir |
| Almutairi, N. [61] | 2022 | Kuwait | 5 | mean age=37 | mRNA | Pain,vesicles, neuralgia / VZV | biopsy | healthy | Acyclovir |
| Chakravorty, S. [62] | 2022 | USA | 10 | 32-73 | 5 Moderna and 5 Pfizer | Fever,Dyspnea,cough,weakness / CMV | PCR | organ transplant | Gancyclovir/valgancyclovir |
| Chew, M. C. [63] | 2022 | Singapore | 6 | mean age=50 | 5 patients pfizer and 1 Sinopharm | uveitis and other eye problems/ CMV AND VZV | PRC | different | prednisolone/ ketorolac/ dexamethasone/ganciclovir/ Clindamycin/ valacyclovir/ Sulfadiazine/ Folinic acid |
| Furer [64] | 2021 | Israel | 6 | 1. 44 2. 56 3. 59 4. 36 5. 38 6. 61 | 1. BNT162b2 mRNA vaccine 2. BNT162b2 mRNA vaccine 3. BNT162b2 mRNA vaccine 4. BNT162b2 mRNA vaccine 5. BNT162b2 mRNA vaccine 6. BNT162b2 mRNA vaccine | 1. Headache, low back pain, vesicular skin rash, and pruritus, consistent with HZ affecting the dermatome L5 2. Malaise, intense headache, and sensation of cold at the left hemicranium, left-sided sever pain of the left eye and forehead, typical rash of HZ, hyperemic conjunctivitis without corneal involvement/HZV 3. Pain and typical HZ vesicular skin rash at the low abdomen, inguinal area, upper thigh, and buttock, without systemic symptoms 4. Pain and typical vesicular skin rash at the abdomen and back 5. Tingling, itching followed by vesicular skin rash typical for HZ at the right breast, without systemic symptoms 6. Typical HZ rash, without systemic symptoms / VZV | 1. Physical examintion 2. Physical and eye examination 3. Physical examination 4. Physical examination 5. Physical examination 6. Physical examination | 1. Sjogren's syndrome, history of varicella 2. Longstanding seropositive RA presented with the first episode of HZ ophthalmicus (HZO), history of varicella 3. Seropositive RA, resistant to multiple biologics and baricitinib, history of varicella 4. Longstanding seropositive RA complicated by interstitial lung disease, history of varicella 5. Undifferentiated CTD and anti-phospholipid syndrome (APS), history of varicella 6. Longstanding seropositive RA, history of varicella | 1. None 2. Acyclovirm lubricant eye drops, and analgesics 3. Valacyclovir 4. Acyclovir 5. Acyclovir 6. Valacyclovir |
| Job [65] | 2022 | India | 7 | 1. 58 2. 51 3. 51 4. 65 5. 26  6. 29 7. 36 | Oxford-AstraZeneca | Five patients reported pain at the site of injection and 3 patients had mild fever lasting <24 h. / VZV | Physical examination | Two among these patients had systemic commorbidities (diabetes and hypertension) | Antivirals, case 4 and case 7 were additionally treated with oral and topical antibiotics |
| Monastirli, A. [66] | 2022 | Greece | 7 | 50-69 | BNT162b2 and moderna(1273) | herpetiform Umbilicated vesicles/ VZV | N.A | healthy | valcyclovir |
| Psichogiou [67] | 2021 | Greece | 7 | 51-94 | Pfizer-BNT 162b2 | Prodromal pain in 5 patients. In all patients a single dermatome was affected; however, two patients developed HZ on the second branch of the trigeminal nerve. / VZV | PCR | None | Oral valacyclovir and medications for neuropathic pain |
| Rodriguez [68] | 2021 | Spain | 5 | 1. 58 2. 47 3. 39 4. 56 5. 41 | BNT162b2 mRNA | 1. Asymptomatic herpetiform umbilicated vesicles, fever, and dervical lymphadenopathy 2. Herpetiform umbilicated vesicles, fever, and dysesthesia 3. Painful herpetiform umbilicated vesicles 4. Herpetiform umbilicated vesicles and dysesthesia 5. Herpetiform umbilicated vesicles and dysesthesia / VZV | 1. PCR positive 2. PCR positive 3. clinical 4. PCR positive 5. clinical | 1.Hypertension | NA |
| Saraiva [69] | 2022 | Portugal | 3 | 1. 79 2. 67 3. 70 | 1. Pfizer, BioNTech (BNT162b2) 2. Moderna 9mRNA-1273) 3. Oxford-AstraZeneca | Painful rash with vesicles and crusts in the skin / VZV | Dermatological examination | 1. Hypertension, dyslipidemia, gout, and benign prostatic hypertrophy 2. Hypertension, dyslipidemia, diabetes mellitus type 2, chronic bronchitis, osteoporosis, and depression 3. Hypertension, dyslipidemia, meningioma, and mammary carcinoma | 1. Gabapentin 2. Brivudine and Diclofenac 3. Brivudine and Ibuprofen |

**Table S5.** The results of quality assessment for observational studies.

| **Author** | **1. Representativeness of the exposed cohort** | **2. Selection of the non exposed cohort** | **3. Ascertainment of exposure** | **4. Demonstration that outcome of interest was not present at start of study** | **5. Comparability** | **6. Assessment of outcome** | **7. Was follow-up long enough for outcomes to occur** | **8. Adequacy of follow up of cohorts** | **Total** |
| --- | --- | --- | --- | --- | --- | --- | --- | --- | --- |
| Birabaharan, M. | 1 | 1 | 1 | 1 | 1 | 1 | 1 | 1 | 8 |
| Català, A. | 1 | 0 | 1 | 1 | 0 | 1 | 1 | 1 | 6 |
| Cebeci Kahraman, F. | 1 | 0 | 1 | 1 | 0 | 0 | 1 | 1 | 5 |
| Hertel, M. | 1 | 1 | 1 | 1 | 1 | 1 | 1 | 1 | 8 |
| Lee, T. J. | 1 | 0 | 1 | 1 | 0 | 0 | 1 | 1 | 5 |
| Préta, L. H. | 1 | 1 | 1 | 1 | 1 | 1 | 1 | 1 | 8 |
| Machado, P. M. | 0 | 1 | 1 | 1 | 0 | 1 | 1 | 1 | 6 |
| Gringeri, M. | 1 | 1 | 1 | 1 | 1 | 1 | 1 | 1 | 8 |
| Florea, A. | 1 | 1 | 1 | 1 | 1 | 1 | 1 | 1 | 8 |
| Fathy, R. A. | 1 | 0 | 1 | 1 | 0 | 0 | 1 | 1 | 5 |
| Chen, J. | 0 | 1 | 1 | 1 | 0 | 1 | 1 | 1 | 6 |

**Table S6.** The results of quality assessment for case reports.

| **Author** | **1. Demography** | **2. History** | **3. Clinical condition** | **4. Diagnose** | **5. Treatment** | **6. Post Treatment** | **7. adverse events** | **8. takeaway lesson** | **Total** |
| --- | --- | --- | --- | --- | --- | --- | --- | --- | --- |
| Abu-Rumeileh, S. | 1 | 1 | 1 | 1 | 1 | 1 | 1 | 0 | 7 |
| Aksu | 1 | 1 | 1 | 0 | 1 | 1 | 0 | 1 | 6 |
| Al-Dwairi, R. A. | 1 | 1 | 1 | 0 | 1 | 1 | 0 | 1 | 6 |
| Algaadi | 1 | 1 | 1 | 0 | 1 | 0 | 1 | 0 | 5 |
| Alkhalifah | 1 | 1 | 1 | 1 | 1 | 0 | 0 | 1 | 6 |
| Ardalan | 1 | 1 | 1 | 0 | 1 | 1 | 0 | 1 | 6 |
| Arora | 1 | 1 | 1 | 0 | 1 | 1 | 0 | 1 | 6 |
| Atiyat | 1 | 1 | 1 | 0 | 1 | 1 | 1 | 1 | 7 |
| Buranasakda | 1 | 1 | 1 | 1 | 1 | 1 | 0 | 1 | 7 |
| Chiu | 1 | 0 | 1 | 0 | 1 | 0 | 1 | 1 | 5 |
| Daouk, S. K. | 1 | 1 | 1 | 1 | 1 | 0 | 0 | 1 | 6 |
| Dermawan, A. | 1 | 1 | 1 | 1 | 1 | 1 | 1 | 1 | 8 |
| Eid | 1 | 1 | 1 | 0 | 0 | 0 | 1 | 1 | 5 |
| Fukuoka | 1 | 1 | 1 | 0 | 1 | 0 | 1 | 1 | 6 |
| Girardin, F. R. | 1 | 1 | 1 | 0 | 0 | 0 | 1 | 1 | 5 |
| Herzum, A. | 1 |  | 1 | 1 | 0 | 0 | 0 | 1 | 4 |
| Jeong | 1 | 1 | 1 | 0 | 0 | 1 | 1 | 1 | 6 |
| Jiang, Z. H. | 1 | 1 | 1 | 1 | 1 | 1 | 0 | 0 | 6 |
| Kerr | 1 | 1 | 1 | 1 | 1 | 1 | 0 | 1 | 7 |
| Kluger | 1 | 1 | 0 | 0 | 1 | 1 | 1 | 1 | 6 |
| Koh | 1 | 1 | 1 | 1 | 1 | 1 | 0 | 1 | 7 |
| Li | 1 | 1 | 1 | 0 | 1 | 1 | 1 | 0 | 6 |
| Lim | 1 | 1 | 1 | 0 | 1 | 1 | 1 | 0 | 6 |
| Lin, T. Y. | 1 | 1 | 1 | 1 | 1 | 1 | 0 | 1 | 7 |
| Lo | 1 | 1 | 0 | 1 | 1 | 1 | 0 | 1 | 6 |
| Lv, Y. | 1 | 1 | 1 | 1 | 1 | 0 | 1 | 1 | 7 |
| Maldonado | 1 | 1 | 1 | 0 | 1 | 1 | 1 | 1 | 7 |
| Maranini | 1 | 1 | 1 | 0 | 1 | 0 | 0 | 1 | 5 |
| Maruki | 1 | 1 | 1 | 1 | 1 | 1 | 1 | 1 | 8 |
| Medhat, R. | 1 | 1 | 1 | 1 | 1 | 1 | 0 | 1 | 7 |
| Mishra | 1 | 1 | 1 | 0 | 1 | 1 | 1 | 0 | 6 |
| Muhie | 1 | 1 | 1 | 0 | 1 | 0 | 0 | 1 | 5 |
| Munasinghe | 1 | 1 | 1 | 1 | 1 | 1 | 1 | 0 | 7 |
| Nastro | 1 | 1 | 1 | 1 | 0 | 1 | 0 | 1 | 6 |
| Nishimoto | 1 | 1 | 1 | 1 | 1 | 0 | 1 | 1 | 7 |
| Ota | 1 | 1 | 1 | 0 | 1 | 0 | 1 | 1 | 6 |
| Ozdemir | 1 | 0 | 1 | 0 | 1 | 1 | 0 | 1 | 5 |
| Palanivel | 1 | 0 | 1 | 0 | 1 | 1 | 0 | 1 | 5 |
| Papasavvas | 1 | 1 | 1 | 0 | 1 | 0 | 1 | 1 | 6 |
| Pedrazini | 1 | 1 | 1 | 0 | 1 | 1 | 1 | 1 | 7 |
| Plub | 1 | 1 | 1 | 1 | 1 | 1 | 1 | 1 | 8 |
| Poudel, S. | 1 | 1 | 1 | 0 | 1 | 0 | 0 | 1 | 5 |
| Said | 1 | 1 | 0 | 1 | 1 | 0 | 1 | 1 | 6 |
| Sangoram, R. | 1 | 1 | 1 | 1 | 1 | 1 | 0 | 1 | 7 |
| Santovito | 1 | 1 | 1 | 0 | 1 | 0 | 1 | 1 | 6 |
| Singh, J. | 1 | 1 | 1 | 0 | 1 | 1 | 0 | 1 | 6 |
| Tang, L. | 1 | 1 | 0 | 1 | 1 | 0 | 1 | 1 | 6 |
| Tang, W. R. | 1 | 1 | 1 | 1 | 1 | 1 | 0 | 1 | 7 |
| Tanizaki, R. | 1 | 1 | 1 | 1 | 1 | 1 | 1 | 1 | 8 |
| Thimmanagari | 1 | 1 | 1 | 0 | 0 | 1 | 1 | 1 | 6 |
| Thonginnetra, S. | 1 | 1 | 1 | 0 | 1 | 1 | 0 | 0 | 5 |
| Tripathy, D. M. | 1 | 1 | 1 | 0 | 1 | 1 | 0 | 1 | 6 |
| Vallianou | 1 | 1 | 1 | 0 | 1 | 1 | 1 | 1 | 7 |
| Van Dam | 1 | 1 | 1 | 0 | 0 | 0 | 1 | 1 | 5 |
| Wang, C. S. | 1 | 1 | 1 | 0 | 1 | 1 | 0 | 1 | 6 |
| You, I. C. | 1 | 1 | 1 | 1 | 1 | 1 | 1 | 1 | 8 |
| Zhang, L. W. | 1 | 1 | 1 | 0 | 1 | 1 | 0 | 1 | 6 |
| Zheng | 1 | 0 | 1 | 1 | 1 | 1 | 0 | 1 | 6 |

**Table S7.** The results of quality assessment for case series.

| **Author** | **inclusion criteria** | **standard measurment** | **valid methods** | **consecutive inclusion** | **complete inclusion** | **Demographics** | **clinical information** | **follow-up outcome** | **sites of demographic information** | **statistical analyses** | **total** |
| --- | --- | --- | --- | --- | --- | --- | --- | --- | --- | --- | --- |
| Alkwikbi | 1 | 1 | 0 | 0 | 0 | 1 | 1 | 1 | 0 | 0 | 5 |
| Furer | 1 | 1 | 0 | 1 | 1 | 1 | 1 | 1 | 1 | 0 | 8 |
| Job | 1 | 1 | 0 | 1 | 0 | 1 | 1 | 0 | 1 | 0 | 6 |
| Psichogiou | 1 | 1 | 1 | 1 | 1 | 1 | 1 | 1 | 1 | 1 | 10 |
| Rodriguez | 1 | 0 | 0 | 1 | 1 | 1 | 1 | 0 | 1 | 1 | 7 |
| Saraiva | 1 | 1 | 0 | 0 | 0 | 1 | 1 | 1 | 0 | 1 | 6 |
| Chew, M. C. | 1 | 1 | 1 | 0 | 1 | 1 | 1 | 1 | 1 | 1 | 9 |
| Monastirli, A. | 1 | 1 | 0 | 0 | 1 | 1 | 1 | 1 | 0 | 0 | 6 |
| Almutairi, N. | 1 | 1 | 1 | 1 | 1 | 1 | 1 | 0 | 1 | 1 | 9 |
| Chakravorty, S. | 1 | 1 | 1 | 1 | 1 | 1 | 1 | 1 | 0 | 1 | 9 |

1. Abu-Rumeileh, S., et al., *Varicella zoster virus-induced neurological disease after COVID-19 vaccination: a retrospective monocentric study.* Journal of neurology, 2022. **269**(4): p. 1751-1757.

2. Al-Dwairi, R.A., et al., *Reactivation of Herpes Simplex Keratitis on a Corneal Graft Following SARS-CoV-2 mRNA Vaccination.* Med Arch, 2022. **76**(2): p. 146-148.

3. Algaadi, S.A., *Herpes zoster after COVID-19 vaccine: A case report.* Pakistan Journal of Medical and Health Sciences, 2021. **15**(3): p. 1165-1166.

4. Alkhalifah, M.I., et al., *Herpes Simplex Virus Keratitis Reactivation after SARS-CoV-2 BNT162b2 mRNA Vaccination: A Report of Two Cases.* Ocul Immunol Inflamm, 2021. **29**(6): p. 1238-1240.

5. Ardalan, M., et al., *Herpes-like skin lesion after AstraZeneca vaccination for COVID-19: A case report.* Clin Case Rep, 2021. **9**(10): p. e04883.

6. Arora, P., et al., *Herpes zoster after inactivated COVID-19 vaccine: A cutaneous adverse effect of the vaccine.* J Cosmet Dermatol, 2021. **20**(11): p. 3389-3390.

7. Atiyat, R., et al., *Varicella-Zoster Virus Reactivation in AIDS Patient After Pfizer-BioNTech COVID-19 Vaccine.* Cureus, 2021. **13**(12): p. e20145.

8. Aksu, S.B. and G.Z. Öztürk, *A rare case of shingles after COVID-19 vaccine: is it a possible adverse effect?* Clin Exp Vaccine Res, 2021. **10**(2): p. 198-201.

9. Buranasakda, M., et al., *Varicella zoster meningitis following COVID-19 vaccination: a report of two cases.* Int J Infect Dis, 2022.

10. Chiu, H.H., et al., *Herpes zoster following COVID-19 vaccine: a report of three cases.* Qjm, 2021. **114**(7): p. 531-532.

11. Daouk, S.K., et al., *Zoster Meningitis in an Immunocompetent Child after COVID-19 Vaccination, California, USA.* Emerging infectious diseases, 2022. **28**(7): p. 1523-1524.

12. Dermawan, A., et al., *Acute herpes zoster radiculopathy mimicking cervical radiculopathy after ChAdOx1 nCoV-19/AZD1222 vaccination.* BMJ Case Rep, 2022. **15**(4).

13. Eid, E., et al., *Herpes zoster emergence following mRNA COVID-19 vaccine.* J Med Virol, 2021. **93**(9): p. 5231-5232.

14. Fukuoka, H., et al., *Oral Herpes Zoster Infection Following COVID-19 Vaccination: A Report of Five Cases.* Cureus, 2021. **13**(11): p. e19433.

15. Girardin, F.R., et al., *Multifocal lymphadenopathies with polyclonal reactions primed after EBV infection in a mRNA-1273 vaccine recipient.* Swiss Med Wkly, 2022. **152**: p. w30188.

16. Herzum, A., et al., *Epstein-Barr virus reactivation after COVID-19 vaccination in a young immunocompetent man: a case report.* Clin Exp Vaccine Res, 2022. **11**(2): p. 222-225.

17. Jeong, J., *Vestibular neuritis after COVID-19 vaccination.* Human Vaccines and Immunotherapeutics, 2021. **17**(12): p. 5126-5128.

18. Jiang, Z.H., et al., *Disseminated and localised herpes zoster following Oxford-AstraZeneca COVID-19 vaccination.* Indian journal of dermatology, venereology and leprology, 2022. **88**(3): p. 445.

19. Kerr, C., et al., *Zoster meningitis in an immunocompetent young patient post first dose of BNT162b2 mRNA COVID-19 vaccine, a case report.* IDCases, 2022. **27**: p. e01452.

20. Kluger, N., T. Klimenko, and S. Bosonnet, *Herpes simplex, herpes zoster and periorbital erythema flares after SARS-CoV-2 vaccination: 4 cases.* Ann Dermatol Venereol, 2022. **149**(1): p. 58-60.

21. Koh, S., et al., *Varicella Zoster Virus Reactivation in Central and Peripheral Nervous Systems Following COVID-19 Vaccination in an Immunocompetent Patient.* J Clin Neurol, 2022. **18**(1): p. 99-101.

22. Li, S.Q., et al., *Herpetic Keratitis Preceded by COVID-19 Vaccination.* Vaccines, 2021. **9**(12).

23. Lim, Z.V., J. Kanagalingam, and Y.K. Heng, *Response to "Varicella-zoster virus reactivation after SARS- CoV-2 BNT162b2 mRNA vaccination: Report of 5 cases".* JAAD Case Rep, 2022.

24. Lin, T.Y., et al., *Hemophagocytic Lymphohistiocytosis Following BNT162b2 mRNA COVID-19 Vaccination.* Vaccines (Basel), 2022. **10**(4).

25. Lo, T., et al., *Varicella Zoster Reactivation Causing Acute Retinal Necrosis following mRNA COVID-19 Vaccination in a Young Immunocompetent Man.* Ocul Immunol Inflamm, 2022: p. 1-4.

26. Lv, Y. and Y. Chang, *Cytomegalovirus Proctitis Developed after COVID-19 Vaccine: A Case Report and Literature Review.* Vaccines (Basel), 2022. **10**(9).

27. Maldonado, M.D. and J. Romero-Aibar, *The Pfizer-BNT162b2 mRNA-based vaccine against SARS-CoV-2 may be responsible for awakening the latency of herpes varicella-zoster virus.* Brain Behav Immun Health, 2021. **18**: p. 100381.

28. Maranini, B., et al., *Herpes zoster infection following mRNA COVID-19 vaccine in a patient with ankylosing spondylitis.* Reumatismo, 2021. **73**(3).

29. Maruki, T., et al., *A case of varicella zoster virus meningitis following BNT162b2 mRNA COVID-19 vaccination in an immunocompetent patient.* Int J Infect Dis, 2021. **113**: p. 55-57.

30. Medhat, R., et al., *Varicella-Zoster Virus (VZV) Meningitis in an Immunocompetent Adult after BNT162b2 mRNA COVID-19 Vaccination: A Case Report.* Int J Infect Dis, 2022. **119**: p. 184-186.

31. Mishra, S.B., et al., *Reactivation of varicella zoster infection presenting as acute retinal necrosis post COVID 19 vaccination in an Asian Indian male.* Eur J Ophthalmol, 2021: p. 11206721211046485.

32. Muhie, O.A., et al., *Herpes Zoster Following Covaxin Receipt.* Int Med Case Rep J, 2021. **14**: p. 819-821.

33. Munasinghe, B.M., et al., *Reactivation of varicella-zoster virus following mRNA COVID-19 vaccination in a patient with moderately differentiated adenocarcinoma of rectum: A case report.* SAGE Open Med Case Rep, 2022. **10**: p. 2050313x221077737.

34. Nastro, F., et al., *Small vessel vasculitis related to varicella-zoster virus after Pfizer-BioNTech COVID-19 vaccine.* J Eur Acad Dermatol Venereol, 2021. **35**(11): p. e745-e747.

35. Nishimoto, M., N. Sogabe, and M. Hino, *Visceral disseminated varicella zoster virus infection following COVID-19 vaccination in an allogeneic stem cell transplant recipient.* Transpl Infect Dis, 2022. **24**(2): p. e13810.

36. Ota, M., *SARS-CoV-2 mRNA vaccination and subsequent herpes zoster: possible immune reconstitution by mRNA vaccination.* JAAD Case Rep, 2022.

37. Özdemir, A.K., S. Kayhan, and S.K. Çakmak, *Herpes zoster after inactivated SARS-CoV-2 vaccine in two healthy young adults.* J Eur Acad Dermatol Venereol, 2021. **35**(12): p. e846-e847.

38. Palanivel, J.A., *Herpes zoster after COVID-19 vaccination-Can the vaccine reactivate latent zoster virus?* Journal of Cosmetic Dermatology, 2021. **20**(11): p. 3376-3377.

39. Papasavvas, I., C. de Courten, and C.P. Herbort, Jr., *Varicella-zoster virus reactivation causing herpes zoster ophthalmicus (HZO) after SARS-CoV-2 vaccination - report of three cases.* J Ophthalmic Inflamm Infect, 2021. **11**(1): p. 28.

40. Pedrazini, M.C. and M.H. da Silva, *Pityriasis rosea-like cutaneous eruption as a possible dermatological manifestation after Oxford-AstraZeneca vaccine: Case report and brief literature review.* Dermatol Ther, 2021. **34**(6): p. e15129.

41. Plüß, M., et al., *Case Report: Cytomegalovirus Reactivation and Pericarditis Following ChAdOx1 nCoV-19 Vaccination Against SARS-CoV-2.* Front Immunol, 2021. **12**: p. 784145.

42. Poudel, S., et al., *Bell's palsy as a possible complication of mRNA-1273 (Moderna) vaccine against COVID-19.* Ann Med Surg (Lond), 2022. **78**: p. 103897.

43. Ryu, K.J. and D.H. Kim, *Recurrence of Varicella-Zoster Virus Keratitis After SARS-CoV-2 Vaccination.* Cornea, 2022. **41**(5): p. 649-650.

44. Said, J.T., et al., *Disseminated varicella-zoster virus infections following messenger RNA-based COVID-19 vaccination.* JAAD Case Rep, 2021. **17**: p. 126-129.

45. Sangoram, R., et al., *Herpes Simplex Virus 1 Anterior Uveitis following Coronavirus Disease 2019 (COVID-19) Vaccination in an Asian Indian Female.* Ocul Immunol Inflamm, 2022. **30**(5): p. 1260-1264.

46. Santovito, L.S. and G. Pinna, *A case of reactivation of varicella-zoster virus after BNT162b2 vaccine second dose?* Inflamm Res, 2021. **70**(9): p. 935-937.

47. Singh, J., et al., *Herpes simplex virus retinitis following ChAdOx1 nCoV- 19 (Covishield) vaccination for SARS CoV 2: A case report.* Ocul Immunol Inflamm, 2022. **30**(5): p. 1282-1285.

48. Tang, L.V. and Y. Hu, *Hemophagocytic lymphohistiocytosis after COVID-19 vaccination.* J Hematol Oncol, 2021. **14**(1): p. 87.

49. Tang, W.R., et al., *A Case Report of Posttransplant Lymphoproliferative Disorder After AstraZeneca Coronavirus Disease 2019 Vaccine in a Heart Transplant Recipient.* Transplant Proc, 2022. **54**(6): p. 1575-1578.

50. Tanizaki, R. and Y. Miyamatsu, *Zoster sine herpete following BNT162b2 mRNA COVID-19 vaccination in an immunocompetent patient.* IDCases, 2022. **29**: p. e01563.

51. Thimmanagari, K., et al., *Ipsilateral Zoster Ophthalmicus Post COVID-19 Vaccine in Healthy Young Adults.* Cureus, 2021. **13**(7): p. e16725.

52. Thonginnetra, S., P. Limtanyakul, and K. Tawinprai, *Herpes zoster after COVID-19 vaccination in an adolescent.* Dermatology online journal, 2022. **28**(4).

53. Tripathy, D.M., et al., *Postherpetic granulomatous dermatitis and herpes zoster necroticans triggered by Covid-19 vaccination.* Dermatol Ther, 2022. **35**(10): p. e15707.

54. Vallianou, N.G., et al., *Herpes zoster following COVID-19 vaccination in an immunocompetent and vaccinated for herpes zoster adult: A two-vaccine related event?* Metabol Open, 2022. **13**: p. 100171.

55. van Dam, C.S., et al., *Herpes zoster after COVID vaccination.* Int J Infect Dis, 2021. **111**: p. 169-171.

56. Wang, C.S., H.H. Chen, and S.H. Liu, *Pityriasis Rosea-like eruptions following COVID-19 mRNA-1273 vaccination: A case report and literature review.* J Formos Med Assoc, 2022. **121**(5): p. 1003-1007.

57. You, I.C., M. Ahn, and N.C. Cho, *A Case Report of Herpes Zoster Ophthalmicus and Meningitis After COVID-19 Vaccination.* J Korean Med Sci, 2022. **37**(20): p. e165.

58. Zhang, L.W., et al., *Disseminated herpes zoster following inactivated SARS-CoV-2 vaccine in a healthy old man.* European journal of dermatology : EJD, 2022. **32**(3): p. 415-416.

59. Zheng, F., A. Willis, and N. Kunjukunju, *Acute Retinal Necrosis from Reactivation of Varicella Zoster Virus following BNT162b2 mRNA COVID-19 Vaccination.* Ocul Immunol Inflamm, 2022. **30**(5): p. 1133-1135.

60. Alkwikbi, H., et al., *Herpetic Keratitis and Corneal Endothelitis Following COVID-19 Vaccination: A Case Series.* Cureus, 2022. **14**(1): p. e20967.

61. Almutairi, N., et al., *Herpes zoster in the era of COVID 19: A prospective observational study to probe the association of herpes zoster with COVID 19 infection and vaccination.* Dermatologic therapy, 2022. **35**(7): p. e15521.

62. Chakravorty, S., et al., *CMV Infection Following mRNA SARS-CoV-2 Vaccination in Solid Organ Transplant Recipients.* Transplantation direct, 2022. **8**(7): p. e1344.

63. Chew, M.C., et al., *Incidence of COVID-19 Vaccination-Related Uveitis and Effects of Booster Dose in a Tertiary Uveitis Referral Center.* Frontiers in medicine, 2022. **9**: p. 925683.

64. Furer, V., et al., *Herpes zoster following BNT162b2 mRNA COVID-19 vaccination in patients with autoimmune inflammatory rheumatic diseases: a case series.* Rheumatology (Oxford), 2021. **60**(Si): p. Si90-si95.

65. Job, A.M., et al., *Herpes zoster following vaccination with ChAdOx1 nCoV-19 Coronavirus vaccine (recombinant).* Indian journal of public health, 2022. **66**(1): p. 83-85.

66. Monastirli, A., et al., *Herpes Zoster after mRNA COVID-19 Vaccination: A Case Series.* Skinmed, 2022. **20**(4): p. 284-288.

67. Psichogiou, M., et al., *Reactivation of Varicella Zoster Virus after Vaccination for SARS-CoV-2.* Vaccines (Basel), 2021. **9**(6).

68. Rodríguez-Jiménez, P., et al., *Varicella-zoster virus reactivation after SARS-CoV-2 BNT162b2 mRNA vaccination: Report of 5 cases.* JAAD Case Rep, 2021. **12**: p. 58-59.

69. Saraiva, A.L., et al., *Varicella zoster virus reactivation following COVID-19 vaccination: a report of 3 cases.* Fam Pract, 2022.
